# Functional characterization of C-TERMINALLY ENCODED PEPTIDE (CEP) family in *Brassica rapa* L

**DOI:** 10.1080/15592324.2021.2021365

**Published:** 2021-12-30

**Authors:** Ziwen Qiu, Keqing Zhuang, Yiting Liu, Xiaomin Ge, Chen Chen, Songping Hu, Huibin Han

**Affiliations:** a Research Center for Plant Functional Genes and Tissue Culture Technology; College of Bioscience and Bioengineering, Jiangxi Agricultural University, Nanchang, China; b Shaanxi Engineering Research Centre for Conservation and Utilization of Botanical Resources, Xi’an Botanical Garden of Shaanxi Province, Institute of Botany of Shaanxi Province, Xi’an City, Shaanxi Province, China

**Keywords:** CEP peptide, *Brassica rapa*, expression pattern, root growth, H_2_O_2_

## Abstract

The small regulatory C-TERMINALLY ENCODED PEPTIDE (CEP) peptide family plays crucial roles in plant growth and stress response. However, little is known about this peptide family in Brassica species. Here, we performed a systematic analysis to identify the putative *Brassica rapa* L. *CEP* (*BrCEP*) gene family. In total, 27 *BrCEP* genes were identified and they were classified into four subgroups based on the CEP motifs similarity. *BrCEP* genes displayed distinct expression patterns in response to both developmental and several environmental signals, suggesting their broad roles during *Brassica rapa* development. Furthuremore, the synthetic BrCEP3 peptide accelerated *Brassica rapa* primary root growth in a hydrogen peroxide (H_2_O_2_) and Ca^2+^ dependent manner. In summary, our work will provide fundamental insights into the physiological function of CEP peptides during *Brassica rapa* development.

## Introduction

Plant development and stress response are tightly regulated by external and internal signals. Recently, studies demonstrate that hormone-like peptides modulate plant growth in a manner similar to phytohormones.^1,2^ The *Arabidopsis thaliana* genome contains more than 7000 small encoding genes, and 200 of them are likely to encode hormone-like peptides.^2^ The C-TERMINALLY ENCODED PEPTIDE (CEP) peptide family belongs to the post-translationally modified peptides, and they need proteolytic enzymes to cleave into bioactive forms, thus executing their physiological function.^3,4^ Each CEP protein contains a secretory N-terminal signal sequence, a central variable region, and highly conserved CEP motifs at C-terminus.^5–7^ CEP peptides have been reported to play pivotal roles in various plant developmental programs.^8–14^

Members of the CEP family have been uncovered in many plant species.^5–7,9,13–18^ The CEP motif (15 amino acids in length) is the biological form of CEP peptide and application of synthetic CEP motifs have been shown to arrest both primary and lateral root development in *Arabidopsis*,^8,10,12,13^ and nodulation in *Medicago truncatula*.^19,20^ In addition, *CEP* genes expression is significantly induced by various abiotic stress, indicating that they also play critical roles in multiple abiotic stress responses.^5,16,21^ Indeed, *Arabidopsis* CEP5 peptide regulates osmotic and drought stress response via interfering with auxin signaling.^21^

CEP peptides also play vital roles in plant nitrogen (N) uptake and transport.^22–24^ Under nitrogen starvation conditions, CEP peptides are transported from N rich side to N deficiency side, and are perceived by the receptor C-TERMINALLY ENCODED PEPTIDE RECEPTOR (CEPR) to upregulate nitrate transporter expression, resulted in N acquisition in roots.^22^ The two identified shoot-derived polypeptides, CEP DOWNSTREAM 1 (CEPD1) and CEPD2, also act as long-distance mobile signals to upregulate the transcription of nitrate transporter genes, thus mediate systemic N-demand signaling downstream of the CEP-CEPR signaling.^23^ CEPD-LIKE2 (CEPDL2) is a leaf-derived signal to regulate N uptake and transport. When roots are unable to obtain enough N to meet shoots N demand, *CEPDL2* expression is significantly upregulated in a CEP-CEPRs dependent manner, CEPDL2 then regulates N uptake and transport via upregulating the transcription of N transporters.^24^

Despite the essential role of CEP peptides in plant development, nitrogen signaling and stress responses, the physiological roles of CEP peptide in Brassica species, however, is poorly understood. Therefore, we selected *Brassica rapa* L., as a model plant with high economic impact to explore the potential roles of CEP peptide family. In this study, we performed a genome-wide search of putative *CEP* genes in *Brassica rapa* and studied their tissue-specific expression patterns as well as their expression in response to environmental stress. The synthetic BrCEP peptides further demonstrate their critical roles in *Brassica rapa* root development. Overall, our work will extend our understanding of the pivotal roles of small regulatory peptides in crop plants growth and their adaptions to the adverse environments.

## Materials and methods

### Identification of the BrCEP gene family

To identify the putative *BrCEP* genes, the full-length sequences of 15 CEP proteins from *Arabidopsis*^5,6^ were used to perform BLASTP against the *Brassica rapa* genome data base (https://phytozome.jgi.doe.gov/pz/portal.html, *Brassica rapa* FPsc V1.3). The identified hits were used as a new query to conduct BLASTP against the same database to avoid any missed BrCEP proteins. The BLASTP was performed repeatedly until no additional novel proteins were found. All the identified BrCEP proteins were aligned with the *Arabidopsis* CEP proteins, and proteins with a similar CEP domain were defined as BrCEP proteins.^5–7^

### BrCEP protein features analysis

SignalP 5.0 (http://www.cbs.dtu.dk/services/SignalP/), the TargetP (http://www.cbs.dtu.dk/services/TargetP), iPSORT (http://ipsort.hgc.jp) and SecretomeP (http://www.cbs.dtu.dk/services/SecretomeP-2.0) were used to predict N-terminal signal peptides of each BrCEP protein. Multiple Expectation Maximization for Motif Elicitation (MEME) was performed to discover the CEP motifs (http://meme.nbcr.net/meme/cgi-bin/meme.cgi).^25^ Weblogo (http://weblogo.berkeley.edu/logo.cgi) was conducted to determinate BrCEP proteins domain features.^26^ Average of isoelectric points and molecular weight of BrCEP proteins were analyzed via the ExPASy Proteomics Server tool (https://web.expasy.org/compute_pi/).

### Genomic organization and chromosome localization of BrCEPs

The genomic sequences and corresponding coding sequences (CDS) of the *BrCEP*s were download. The genomic organization of the *BrCEP* genes were performed via gene structure display server with default settings (http://gsds.cbi.pku.edu.cn).^27^ The distribution of the *BrCEP* genes on the chromosome were conducted via the MG2C online software with default settings (http://mg2c.iask.in/mg2c_v2.0/).

### Identification of cis-acting regulatory elements

Upstream region (1500 bp) of the BrCEP genes were used to perform the cis-acting regulatory element analysis at PlantCARE website (http://bioinformatics.psb.ugent.be/webtools/plantcare/html/).^28^

### Alignment and phylogenetic analysis

Multiple alignments were performed using ClustalX,^29^ then refined and displayed using GeneDoc (http://www.psc.edu/biomed/genedoc). Phylogenetic trees were constructed by MEGA X using the conserved CEP domains or the full length of CEP proteins by the Maximum likelihood method.^30^ Bootstrap analysis was conducted with 1000 replicates to verify the significance of nodes.

### Expression patterns of BrCEPs

4 microarray datasets (GSE73963, GSE43245, GSE123654 and GSE55264) were collected from Gene Expression Omnibus database (GEO) at NCBI website to determine the *BrCEP* genes expression in a given developmental context or under environmental stress. Transcription abundances based on RNA-Seq data were normalized and calculated as log2 expression value. Heatmaps were generated based on the expression profiles via the online tool (https://www.omicstudio.cn/tool/4).

### Synthesis of the BrCEP peptides and chemicals

The 15 amino acids motif of the BrCEP3 (DFRPTTPGHSPGIGH) and BrCEP26 (TFRPTVPGHSPGIGH) were sent for synthesis by DGpeptide company (http://www.dgpeptides.com/) with a purity higher than 90%. All peptides were dissolved in distilled water to a concentration of 10 mM and were aliquoted to avoid repeated freeze-thaw cycles, and were stored at -20°C. Catalase (CAT), diphenylene iodonium (DPI), hydrogen peroxide (H_2_O_2_) and lanthanum chloride were obtained from Macklin company (http://www.macklin.cn/).

### Plant Material and Growth Conditions

The cultivar *Brassica rapa* seeds were bought from Beijing Dongsheng company (http://dsseed.fsfseed.com/Index.asp). All seeds were washed with 70% ethanol for 1 minute. Then seeds were sterilized with 0.1% HgCl_2_ for 8 minutes, afterward seeds were washed with distilled water for 5 times. The sterilized seeds were sown on 1% agar plate containing half-strength Murashige & Skoog medium (½MS). The seedlings were grown vertically at 21°C under a 16 h: 8 h (light: dark photoperiod) with a light intensity 112 μmol m^−2^ sec^−1^ in a plant growth chamber.

### Pharmacological treatments and root length quantification

After germination (roughly 2 days), the seedlings with a similar root length were transferred to new plates supplied with BrCEP3 (1 μM), BrCEP26 (1 μM), CAT (1000 units), DPI (10 μM) and lanthanum chloride (500 μM), H_2_O_2_ (1 mM) or the combination with BrCEP3 peptide, and the seedlings were cultured for another 4 days in the same growth chamber. Plates were imaged via the EPSON V370 scanner. The root length was quantified via ImageJ. At least three independent experiment was performed to yield the similar results. For each treatment, 10–15 roots were quantified.

### H_2_O_2_ staining

The DAB (3,3′-diaminobenzidine, BIORIGIN company http://www.biorigin.ltd/, BN20341) staining kit was used to quantify H_2_O_2_ level in *Brassica rapa* roots, the staining was performed follow the instructions. Briefly, *Brassica rapa* seedlings treated with mock or 1 μM BrCEP3 peptide for 4 days were stained with 1× DAB buffer (0.1% DAB in 1× PBS buffer, pH 7.4) for 3–5 minutes at room temperature, then the staining was terminated by washing seedlings with 1× PBS buffer (0.01 M, pH 7.4) for 3 times. The samples were mounted on slides immediately, and were captured with an inverted BDS 400 microscopy (http://www.cnoptec.com/). Three independent biological repeats with 10 seedlings for each treatment were performed to yield a similar result. The H_2_O_2_ level was quantified via ImageJ.

### Statistical analysis

All statistical analysis was performed using the one-way ANOVA test with a significant difference (* *P* < .05, ** *P* < .01, *** *P* < .001).

## Results

### *Genome-wide search and characterization of* BrCEP *gene family*

**Figure 1.**
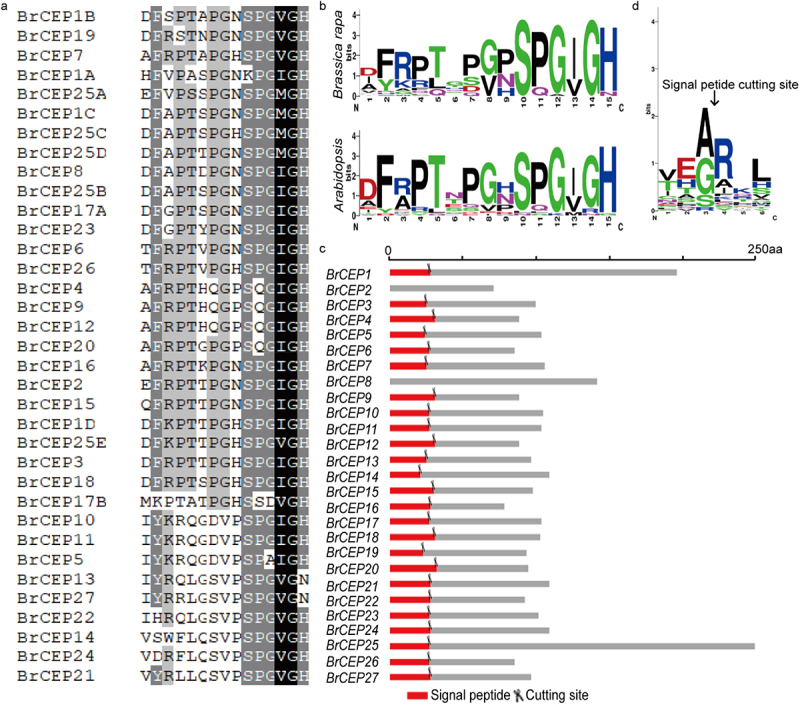
Identification of *BrCEP* gene family. (a) Alignment of the 27 *BrCEP* CEP motifs. (b) WebLogo representation of the conserved BrCEP and AtCEP motifs. (c) Signal peptide of BrCEP proteins. The red boxes represent signal peptides, and the scissors represent the predicted signal peptide cleavage posotion. (d) WebLogo representation of the signal cleavage sites.

We used 15 *Arabidopsis* CEP (AtCEP) full protein sequences to perform BLASTP against the *Brassica rapa* genome database.^5,6^ As a result, 27 putative *BrCEP* genes were identified, and they displayed similar CEP motifs (Figure 1a, b). The corresponding coding sequence (CDS) of *BrCEP* genes ranged from 210 base pairs (*BrCEP2*) to 732 base pairs (*BrCEP25*) with the protein size ranging from 69 to 243 amino acids in length, respectively. The molecular weight and isoelectric point of BrCEP proteins ranged from 7902.06 Da (BrCEP2) to 26040.83 Da (BrCEP25) and from 4.71 (BrCEP5) to 10.82 (BrCEP14), respectively (Table S1).

**Figure 2.**
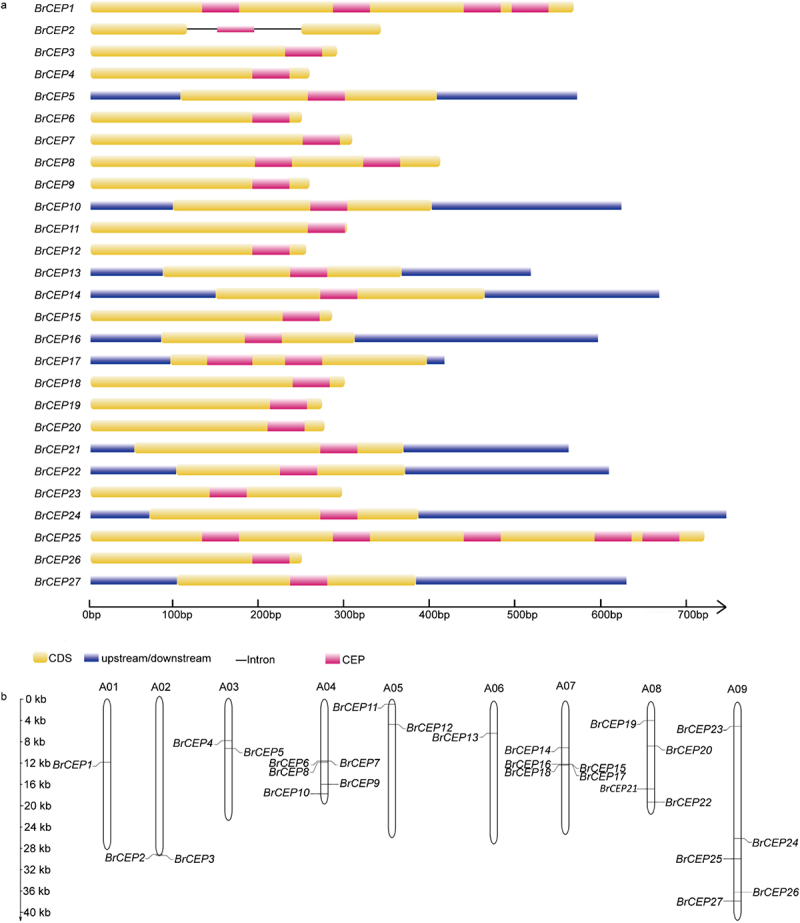
Genomic organization and chromosome localization of *BrCEP* genes. (a) Gene structure of *BrCEP* genes. (b) Distribution of *BrCEP* genes on *Brassica rapa* chromosomes.

We next performed a detailed characterization of the BrCEP proteins, including CEP motif distribution, N-terminal signal peptide, genomic organization and chromosome localization. Based on the MEME analysis, BrCEP1 and BrCEP25 displayed four and five CEP domains, respectively; BrCEP8 contained two identical CEP domains; BrCEP17 contained two CEP domains (Figure 1a; Figure 2a; Table S1). We next searched the presence and location of the putative N-terminal signal peptide cleavage sites in each BrCEP protein (Figure 1c; Table S2). Based on the prediction, it is likely that the cleavage site occurred at a conserved arginine site (Figure 1d; Table S2), which also has been shown for apple CEP proteins.^15^ However, we could not find any cleavage site for BrCEP2 and BrCEP8 proteins, this may due to the limitations of the software.

We also analyzed the genomic organization and chromosome localization of *BrCEP* genes. Only *BrCEP2* contained two introns, the rest of the *BrCEP*s lack introns (Figure 2a). *BrCEP*s scattered over on 9 chromosomes (Figure 2b). The chromosome A01 and A06 only contained one *BrCEP* gene although some clustering was observed in other chromosomes (Figure 2b). For example, *BrCEP6, BrCEP7* and *BrCEP8* were organized sequentially in tandem on chromosome A04. A similar cluster was also observed for *BrCEP15, BrCEP16, BrCEP17* and *BrCEP18* on chromosome A07. Notably, the clustered *BrCEP*s showed low sequence similarity, and the CEP motifs were not totally identical, suggesting that these genes might not arise from recent tandem duplication events (Figure 1; Figure 2). Interestingly, some *BrCEP*s localized on different chromosomes encoded identical CEP domains, for instance, the CEP motifs of the pairs of *BrCEP4*/*BrCEP9*/*BrCEP12, BrCEP10*/*BrCEP11* were identical (Figure 1; Table S1). These results suggested that genome-scale duplication of *BrCEP* genes occurred in different regions of *Brassica rapa* chromosomes.

Taken together, our analysis showed that BrCEP proteins displayed several features similar to the known CEP proteins, including C-terminal conserved CEP motifs, N-terminal signal and a central variable region (Figure 1; Figure S1; Table S1).

### Phylogenetic analysis of BrCEP proteins

**Figure 3.**
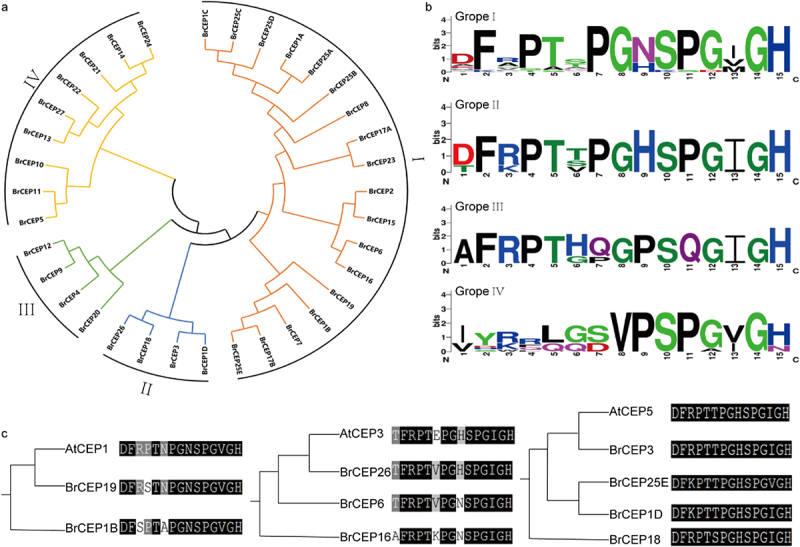
The BrCEP proteins are classified into four subgroups. (a) The phylogenetic tree of BrCEP proteins baesd on the CEP motifs. (b) WebLogo representation of CEP motifs in each subgroups. (c) Comparative alignment of the AtCEP and BrCEP proteins with identical or nearly identical CEP motifs. Each clade contains a known function of the AtCEP protein and their closest BrCEP protein derived from the phylogenetic analysis of all AtCEP and BrCEP CEP motifs (Fig. S3).

CEP motif and full-length sequences of BrCEP proteins were used to build phylogenetic trees. Based on the conversed CEP motifs, the BrCEP proteins were divided into four subgroups (Figure 3a). The CEP motifs of the four subgroups were aligned, which resulted in a consensus sequences supporting for classification of the four groups (Figure 3b). Whereas the multiple CEP domains of BrCEP1 and BrCEP25 proteins were not grouped into the same group due to the divergent sequence (Figure 3a). Notably, the phylogenetic relationship based on the CEP motifs was not well supported when the full-length BrCEP proteins were analyzed, due to the divergent amino acid sequences outside the CEP motifs (Figure S2).

To probe the potential roles of the BrCEP proteins, the phylogenetic relationship between AtCEP proteins and BrCEP proteins were analyzed using either the CEP motifs (Figure S3) or the full-length protein sequences (Figure S4). The BrCEP and AtCEP proteins were grouped into several clades with varying degrees. Notably, some of the BrCEP motif that matched completely or near identical to the known function of the AtCEP peptides (Figure 3c; Figure S5).^5,10–12,21^ For instance, *AtCEP5* and *BrCEP3* encoded an identical CEP motif, suggesting that *BrCEP*s would play essential roles in *Brassica rapa* development.

### BrCEP*s respond to developmental and environmental signals*

The *cis* regulatory elements in the promoter region are important for gene expression regulation, we first performed an analysis to identify the *cis*-acting regulatory elements in *BrCEP* promoter regions. Numbers of *cis*-acting regulatory elements were discovered (Figure S6). The stress-related regulatory elements such as TC-rich repeat, MBS (Drought responsive element), LTR (Low temperature responsive element), and ABRE (ABA responsive element) elements were found in many *BrCEP* genes, further suggesting the potential roles of *BrCEP*s in stresses-related developmental processes. Furthermore, the identified phytohormone-related elements indicating potential crosstalk between CEP peptides and hormones signalings during *Brassica rapa* development.

We next addressed *BrCEP*s expression patterns in *Brassica rapa* tissues. The *BrCEP* genes expression levels in 5 tissues, including stem, root, leaf, sillque and flower were visualized (Figure S7a). The expression patterns of *BrCEP*s were varied in the examined tissues. For example, *BrCEP25* showed highest expression in roots; *BrCEP22* was highly expressed in sillque; *BrCEP16* showed relative high transcription level in flowers. The diverse expression patterns of *BrCEP* genes in various tissues which indicates distinct functions of *BrCEP*s during various aspects of *Brassica rapa* developmental processes.

As many stress-related regulatory elements were found in *BrCEP* promoters (Figure S6), we then examined whether *BrCEP*s expressions were induced by environmental stress. Our analysis showed that *BrCEP*s expression level were differentially regulated by several environmental signals such as drought, circadian rhythm and trace element (Figure S7b, c, d,). The expression levels of *BrCEP* genes were varied in time points upon drought induction (Figure S7b). For example, *BrCEP10* was upregulated after 4 hours and 8 hours drought induction; and *BrCEP11* was upregulated and *BrCEP25* was downregulated after 4 hours drought induction; *BrCEP24* was upregulated after 8 hours drought induction, but it was downregulated after 12 hours drought treatment. The distinct spatio-temporal expression of *BrCEP*s indicates that *BrCEP*s would play distinct roles in various stages of drought response.

Plant circadian rhythm coordinates the complicated cellular responses to multiple and often simultaneous environmental signals, resulting in efficient changes of dynamic metabolic, physiological and defense networks to guarantee plant growth.^31^ A result of the circadian integration of the environmental cues is the activation of the complex cellular response to a given stimulus such as drought response.^31^ *BrCEP*s genes were also differentially induced by circadian rhythms (Figure S7c), indicating that circadian rhythms potentially activate the *BrCEP* expression and stresses-related regulatory networks to coordinate *Brassica rapa* development under unfavorable conditions.^32^

Trace element minerals such as iron (Fe) and zinc (Zn) are essential for plant growth. Zinc (Zn) is a crucial micronutrient and involved in many physiological processes; however, excessive Zn content triggers plant chlorosis, photosynthesis inhibition, resulted in crop growth perturbations.^33^ Our analysis revealed that *BrCEP*s expression was differentially regulated by Zn and Fe (Figure S7d). Under Zn or Fe deficiency conditions, *BrCEP8* and *BrCEP11* was up-regulated. Under Zn excessive condtion, *BrCEP8* was downregulated, however, *BrCEP10* was upregulated. We hence speculated that CEP peptides participate in maintaining Fe and Zn homeostasis during *Brassica rapa* development.

Cadmium (Cd) is ranked the 7th on the list of the top 275 hazardous materials.^34^ Brassica species can absorb most of the Cd from the soil, they are considered as potential candidates for Cd phytoremediation.^35^ Brassica species can tolerate Cd via multiple mechanism, as a result, they can be used to produce biofuel. Notaly, Cd also seriously affect Brassica growth and development. Our analysis showed that *BrCEP*s were induced by Cd treatment, for example, *BrCEP8* and *BrCEP19* were upregulated (Figure S7d). It is likely that *BrCPE*s could help Brassica species to absorb the Cd and improve Brassica tolerance to excess Cd content.

Taken together our analysis obtained from public microarray data suggests that *BrCEP*s respond differentially to both developmental and several environmental signals, indicating that *BrCEP*s may play distinct roles in various *Brassica rapa* developmental processes and adaptations.

### *BrCEP3 and BrCEP26 peptides promote Brassica* rapa *primary root growth*

**Figure 4.**
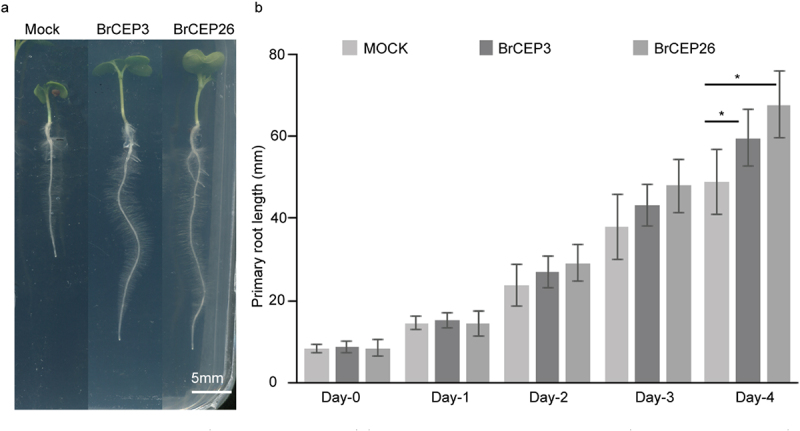
BrCEP3 and BrCEP26 peptide promote *Brassica rapa* primary root growth. (a) Representative images showing the *Brassica rapa* seedlings treated with BrCEP3, BrCEP26 peptide for 4 days. (b) Quantification of 1 μM of BrCEP3 and BrCEP26 peptide treated *Brassica rapa* seedlings primary root length. N = 10–15 seedlings, Error bars are SD, * *P* < .05 was determined by One-way ANOVA. Scare bar = 5 mm.

We synthesized two BrCEP peptides, BrCEP3 and BrCEP26, to further explore their physiological effects on *Brassica rapa* seedling development. After germination, *Brassica rapa* seedlings with a comparable primary root length were transferred to new agar plates supplied with 1 μM of BrCEP3 and BrCEP26 peptides, respectively. The seedlings were grown for another 4 days, and primary root length was quantified. Under our experimental conditions, we observed that both BrCEP3 and BrCEP26 peptides significantly promoted *Brassica rapa* primary root growth (Figure 4).

### Hydrogen peroxide and Ca^2+^ is involved in BrCEP3-mediated root growth

**Figure 5.**
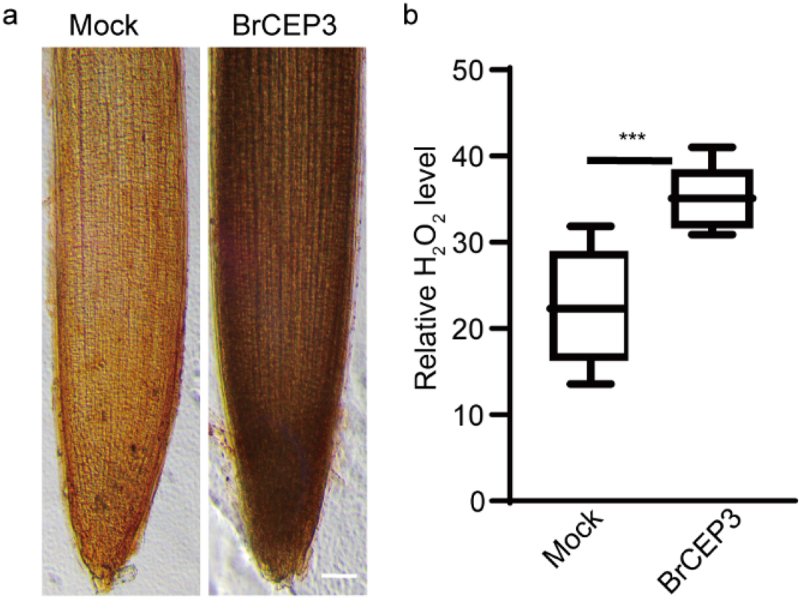
BrCEP3 peptide promotes H_2_O_2_ accumulation in *Brassica rapa* primry roots. (a) Representative images showing ROS level in *Brassica rapa* primary roots treated with mock or 1 μM BrCEP3 peptide for 4 days. (b) Quantification of ROS level. N = 10, *** *P* < .001 was determined by One-way ANOVA test. Data represents mean ± SD. Scare bar = 50 μm.

**Figure 6.**
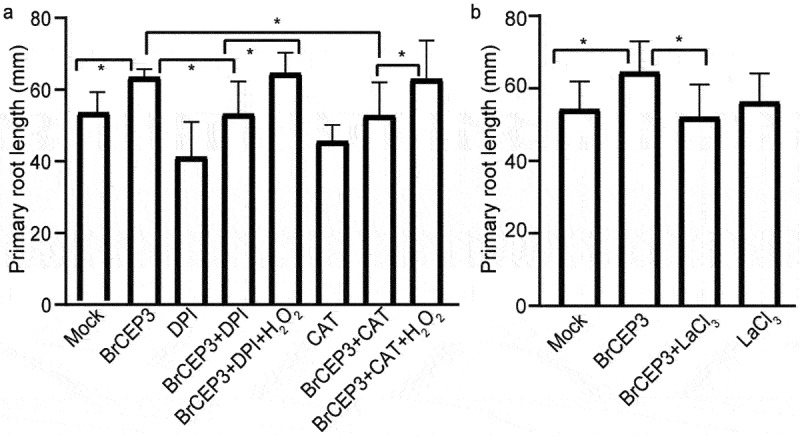
H_2_O_2_ and Ca^2+^ are required for BrCEP3-mediated root growth. Quantification of *Brassica rapa* seedlings primary root length upon treatment of H_2_O_2_ inhibitors (a) and Ca^2+^ inhibitor (b) for 4 days. N = 10–15, * *P* < .05 was determined by One-way ANOVA test. Data represents mean ± SD.

We next addressed the involvement of hydrogen peroxide (H_2_O_2_) and Ca^2+^ in BrCEP3-mediated root growth.^36,37^ We first tested the impact of BrCEP3 peptide on H_2_O_2_ level in *Brassica rapa* primary root. Our DAB staining result showed that BrCEP3 triggered a prominent accumulation of H_2_O_2_ in primary root (Figure 5). Next, we examined the effects of CAT (a H_2_O_2_ scavenger enzyme), and DPI (an inhibitor of NADPH oxidase) on BrCEP3-mediated root growth. DPI prominently inhibited BrCPE3-mediated root growth, whereas CAT slightly repressed BrCEP3 effect on root growth, even the CAT and DPI showed an inhibitory effect on root growth. Notably, exogenous application H_2_O_2_ suppressed CAT and DPI effects (Figure 6a). In addition, when Ca^2+^ channel was blocked by lanthanum chloride (Ca^2+^ channel blocker), BrCEP3-induced root growth promotion was also abolished (Figure 6b). These collective data showed that H_2_O_2_ and Ca^2+^ signaling are necessary for BrCEP3 peptide to facilitate *Brassica rapa* primary root elongation.

## Discussion

In this study, a systematic analysis of the *CEP* gene family in *Brssica rapa* L. was performed. A total of 27 *BrCEP* genes were uncovered, and they displayed similar features to the *CEP* members identified in various species. *BrCEP* genes also exhibited distinct expression patterns in response to both developmental and environmental signals. In addition, H_2_O_2_ and Ca^2+^ were involved in BrCEP3 peptide-mediated *Brassica rapa* root development.

It has been suggested that post-translational modifications mediated by many enzymes are necessary to produce the functional mature peptides.^38–40^ However, how functional mature BrCEP peptides are generated remains elusive. On the other hand, how these modification enzymes affect the BrCEP function and signaling transduction are also unknown. The answers to these fundamental questions will greatly help to understand BrCEP peptides function.

Data obtained from public transcriptome datasets showed that *BrCEP* genes displayed diverse expression patterns in a given tissue or under environmental stress conditions (Fig. S7). Considering only some *BrCEP* genes can be detected from the public expression database, the transcription levels of other *BrCEP* genes require further confirmation.

To our surprise, BrCEP3 and BrCEP26 peptide promoted primary root growth (Figure 4), indicating diverse CEP peptide functions in species. Antagonistic peptide technology has been developed to dissect CLE peptide function,^41,42^ it is likely BrCEP26 peptide maybe an antagonistic form, but it definitely requires careful examinations in future. However, *BrCEP3* encodes an identical CEP motif to *Arabidopsis CEP5* (Figure 3c), hence, it is possible that BrCEP3 peptide may target different downstream signaling networks in *Brassica rapa* to promote primary root growth (Figure 4). Additionally, conserved serine (at position 10) and glycine (at position 14) in the apple MdCEP1 motif are crucial for its function,^15^ thus, it is an open question to examine whether these conserved serines and glycine are also essential for BrCEP peptide function. Nevertheless, how BrCEP peptide is perceived by its putative receptors remains elusive, identification of RLKs in Brassica species will provide novel insights into BrCEP signaling transduction.^43–45^

Reactive oxygen species (ROS) acts as essential signaling molecule to regulate plant root development. When ROS homeostasis is disturbed in root, plants are unable to adjust their growth in the changing environments.^46^ H_2_O_2_ is a kind of ROS, which is generated by the two main NADPH/RBOH oxidases, RBOHD and RBOHF.^47–49^ Our pharmacological experiment showed that H_2_O_2_ is required for BrCEP3-mediated root growth (Figure 6a). How BrCEP3 peptide affects H_2_O_2_ level via RBOHD and RBOHF is elusive. On the other hand, we can not exclude the involvement of other ROS forms, such as nitric oxide (NO) in BrCPE3-mediated root growth. Additionally, Ca^2+^ was also involved in BrCPE3-mediated root growth (Figure 6b). The genetic mutants related to ROS biosynthesis and signaling,^36^ as well as Ca^2+^ signaling^37^ will help to uncover more essential components involved in BrCEP3 peptide signaling pathway.

Environmental stress severely affects crop growth and yield. Therefore, plants have evolved multiple strategies including activation of CEP peptide signaling cascade to adjust their adaptions to the changing environment.^5,16,21^ The induction of *BrCEP*s expression by environmental stress further indicates their roles in *Brassica rapa* adaptions to adverse environments (Figure S7b, c, d). To discover more key participants and the precise mechanisms in BrCEP-mediated stress adaptions is one of fascinating topics in future investigations.

## Supplementary Material

Supplemental Material
