# Supplementary material for: Functional characterization of C-TERMINALLY ENCODED PEPTIDE (CEP) family in Brassica rapa L
Source: Plant Signal Behav. 2021 Dec 30;17(1):2021365. doi: 10.1080/15592324.2021.2021365 (PMC8920145; doi:10.1080/15592324.2021.2021365)
Supplement: Supplemental Material [file KPSB_A_2021365_SM4165.docx]

**Tab S1 Summary of the identifed 27 BrCEP proteins**

| **Gene** | **Locus ID** | **Chr. Position** | **Genomic sequence ( bp)** | **Transcript sequence( bp)** | **CDS sequence (bp)** | **Protein length (AA)** | **Molecular weight（Da）** | **PI** | **CEP motif (15AA)** |
| --- | --- | --- | --- | --- | --- | --- | --- | --- | --- |
| *BrCEP1* | Brara.A02125.1 | A01:13084474~13085049 | 576 | 576 | 576 | 191 | 20551.14 | 9.48 | HFVPASPGNKPGIGH  DFSPTAPGNSPGVGH  DFAPTSPGNSPGMGH  DFKPTTPGHSPGIGH |
| *BrCEP2* | Brara.B03982.1 | A02:32569131~32575100 | 5970 | 210 | 210 | 69 | 7902.06 | 9.03 | EFRPTTPGNSPGIGH |
| *BrCEP3* | Brara.B04005.1 | A02:32754874~32755167 | 294 | 294 | 294 | 97 | 10730.32 | 7.94 | DFRPTTPGHSPGIGH |
| *BrCEP4* | Brara.C01780.1 | A03:8718937~8719197 | 261 | 261 | 261 | 86 | 9607.97 | 9.4 | AFRPTHQGPSQGIGH |
| *BrCEP5* | Brara.C02077.1 | A03:10376572~10377151 | 580 | 580 | 306 | 101 | 11044.62 | 4.71 | IYKRQGDVPSPAIGH |
| *BrCEP6* | Brara.D01395.1 | A04:12707437~12707688 | 252 | 252 | 252 | 83 | 8822.16 | 6.24 | TFRPTVPGNSPGIGH |
| *BrCEP7* | Brara.D01396.1 | A04:12719678~12719989 | 312 | 312 | 312 | 103 | 12151.63 | 10.07 | AFRPTAPGHSPGVGH |
| *BrCEP8* | Brara.D01445.1 | A04:13081166~13081582 | 417 | 417 | 417 | 138 | 15081.15 | 9.22 | DFAPTDPGNSPGIGH  DFAPTDPGNSPGIGH |
| *BrCEP9* | Brara.D02149.1 | A04:17518687~17518947 | 261 | 261 | 261 | 86 | 9549.86 | 9.04 | AFRPTHQGPSQGIGH |
| *BrCEP10* | Brara.D02468.1 | A04:19489223~19489855 | 633 | 633 | 309 | 102 | 11175.61 | 4.79 | IYKRQGDVPSPGIGH |
| *BrCEP11* | Brara.E00176.1 | A05:1008023~1008328 | 306 | 306 | 306 | 101 | 11213.79 | 6.4 | IYKRQGDVPSPGIGH |
| *BrCEP12* | Brara.E00884.1 | A05:5201666~5201926 | 261 | 261 | 261 | 86 | 9634.95 | 9.4 | AFRPTHQGPSQGIGH |
| *BrCEP13* | Brara.F01185.1 | A06:6581107~6581631 | 525 | 525 | 285 | 94 | 10053.56 | 8.54 | IYRQLGSVPSPGVGN |
| *BrCEP14* | Brara.G00767.1 | A07:9672780~9673457 | 678 | 678 | 321 | 106 | 11642.81 | 10.82 | VSWFLQSVPSPGVGH |
| *BrCEP15* | Brara.G01218.1 | A07:13091274~13091561 | 288 | 288 | 288 | 95 | 10271.86 | 9.92 | QFRPTTPGNSPGIGH |
| *BrCEP16* | Brara.G01219.1 | A07:13094957~13095561 | 605 | 605 | 231 | 76 | 7992.33 | 8.98 | AFRPTKPGNSPGIGH |
| *BrCEP17* | Brara.G01220.1 | A07:13099912~13100333 | 422 | 422 | 306 | 101 | 11098.33 | 5.88 | DFGPTSPGNSPGIGH MKPTATPGHSSDVGH |
| *BrCEP18* | Brara.G01224.1 | A07:13148496~13148798 | 303 | 303 | 303 | 100 | 10929.76 | 8.69 | DFRPTSPGHSPGIGH |
| *BrCEP19* | Brara.H00404.1 | A08:3931086~3931361 | 276 | 276 | 276 | 91 | 10161.47 | 8.22 | DFRSTNPGNSPGVGH |
| *BrCEP20* | Brara.H00710.1 | A08:9206875~9207153 | 279 | 279 | 279 | 92 | 10229.89 | 10.36 | AFRPTGPGPSQGIGH |
| *BrCEP21* | Brara.H01955.1 | A08:18072132~18072701 | 570 | 570 | 321 | 106 | 11894.06 | 10.6 | VYRLLQSVPSPGVGH |
| *BrCEP22* | Brara.H02490.1 | A08:20680172~20680789 | 618 | 618 | 273 | 90 | 9622.2 | 7.78 | IHRQLGSVPSPGVGH |
| *BrCEP23* | Brara.I00882.1 | A09:4901177~4901476 | 300 | 300 | 300 | 99 | 10949.36 | 6.02 | DFGPTYPGNSPGIGH |
| *BrCEP24* | Brara.I02898.1 | A09:28097263~28098020 | 758 | 758 | 321 | 106 | 11483.52 | 10.76 | VDRFLQSVPSPGVGH |
| *BrCEP25* | Brara.I03403.1 | A09:32284903~32285634 | 732 | 732 | 732 | 243 | 26040.83 | 8.62 | EFVPSSPGNSPGMGH DFAPTSPGNSPGIGH DFAPTSPGHSPGMGH DFAPTTPGNSPGMGH DFKPTTPGHSPGVGH |
| *BrCEP26* | Brara.I04531.1 | A09:39192889~39193140 | 252 | 252 | 252 | 83 | 8805.1 | 5.66 | TFRPTVPGHSPGIGH |
| *BrCEP27* | Brara.I04854.1 | A09:40971617~40972255 | 639 | 639 | 285 | 94 | 10011.61 | 8.71 | IYRRLGSVPSPGVGN |

**Tab S2 N-terminal cleavage site of the BrCEP proteins**

| **Protein** | **Cleavage position** | **Cleavage sequence** |
| --- | --- | --- |
| BrCEP1 | 26-27 | QEQITTEARKLRKTIG |
| BrCEP2 |  |  |
| BrCEP3 | 24-25 | LFNGFNHGRTLRNMKV |
| BrCEP4 | 30-31 | LHFDPTTAARHAPVVS |
| BrCEP5 | 23-24 | LLISEVPAILGFSMRG |
| BrCEP6 | 26-27 | QEFCSVEGRTLAKST |
| BrCEP7 | 24-25 | FSEFIGEAQGSRLRRH |
| BrCEP8 |  |  |
| BrCEP9 | 30-31 | LHFETTTAARHAPFVS |
| BrCEP10 | 26-27 | TEVPSILGLSTRGNTR |
| BrCEP11 | 26-27 | SIAPSIHGFKMRGITR |
| BrCEP12 | 30-31 | LHFETTTGARHAPVVS |
| BrCEP13 | 24-25 | IVGFVSQSYEARKVVM |
| BrCEP14 | 20-21 | VLAVIIVASPSPVVSR |
| BrCEP15 | 29-30 | HEILPTEARHLGTHRK |
| BrCEP16 | 27-28 | QETHIVEGRPLKSLG |
| BrCEP17 | 26-27 | DKIQYNEARQLQTDGK |
| BrCEP18 | 30-31 | LGFSCVHGRILKNMNV |
| BrCEP19 | 22-23 | LGLAVLHGIQYTEERH |
| BrCEP20 | 30-31 | LHFRTIAAARKSVKVF |
| BrCEP21 | 27-28 | VSPLPVSSRKLLEMKN |
| BrCEP22 | 27-28 | FASQPCEARNVLMPYN |
| BrCEP23 | 26-27 | YKIPYTEARQLRETDS |
| BrCEP24 | 27-28 | ALPSPVSSRKLLEMK |
| BrCEP25 | 26-27 | QAPITTEARKLRKTTG |
| BrCEP26 | 26-27 | QELGSVEGRTLTTSTM |
| BrCEP27 | 27-28 | FVSQSCEARKVLMPYG |

**
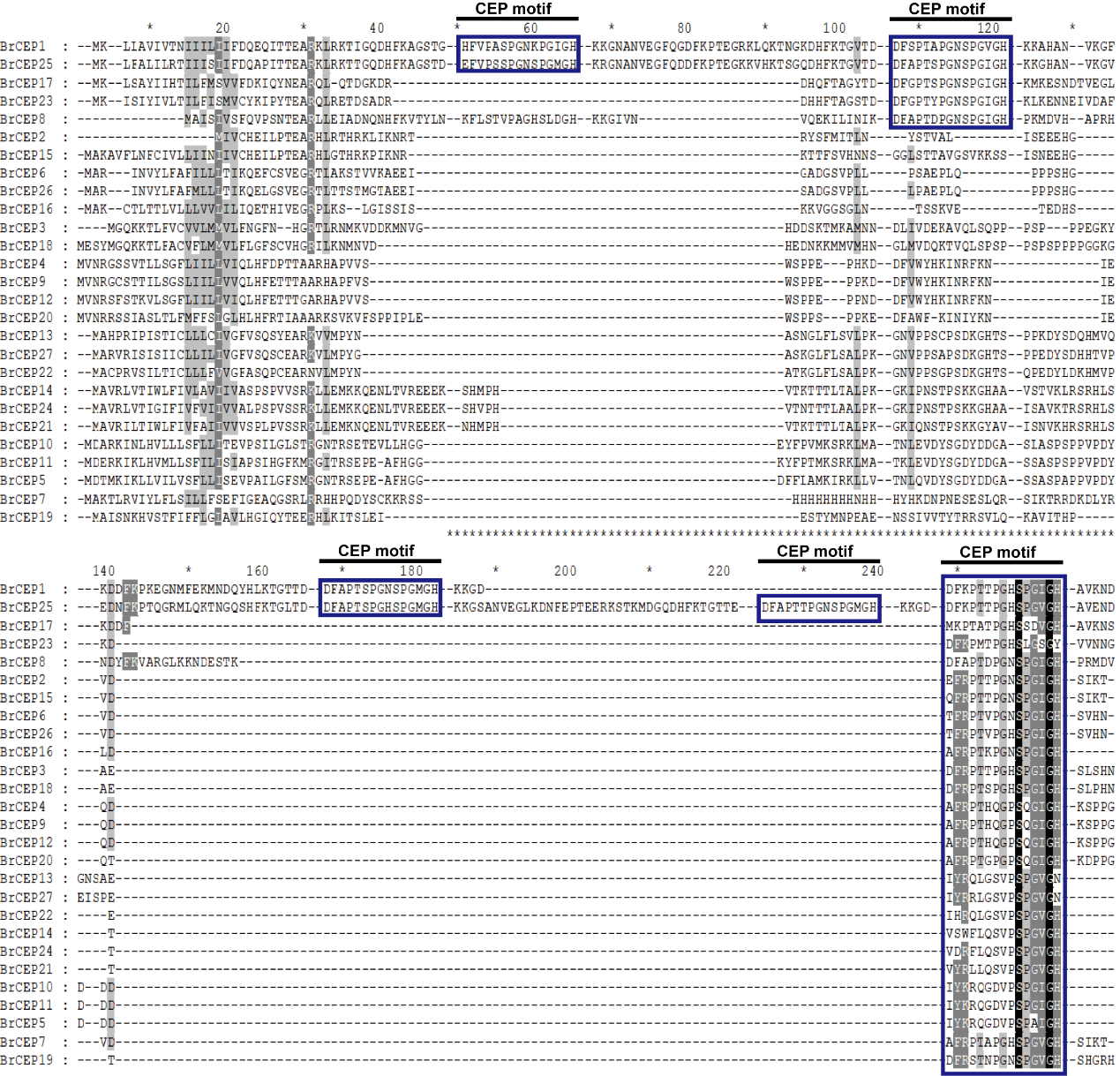
**

**Figure S1.** Protein alignment of full-length BrCEP proteins. The CEP motifs were indicated by the blue box.

**
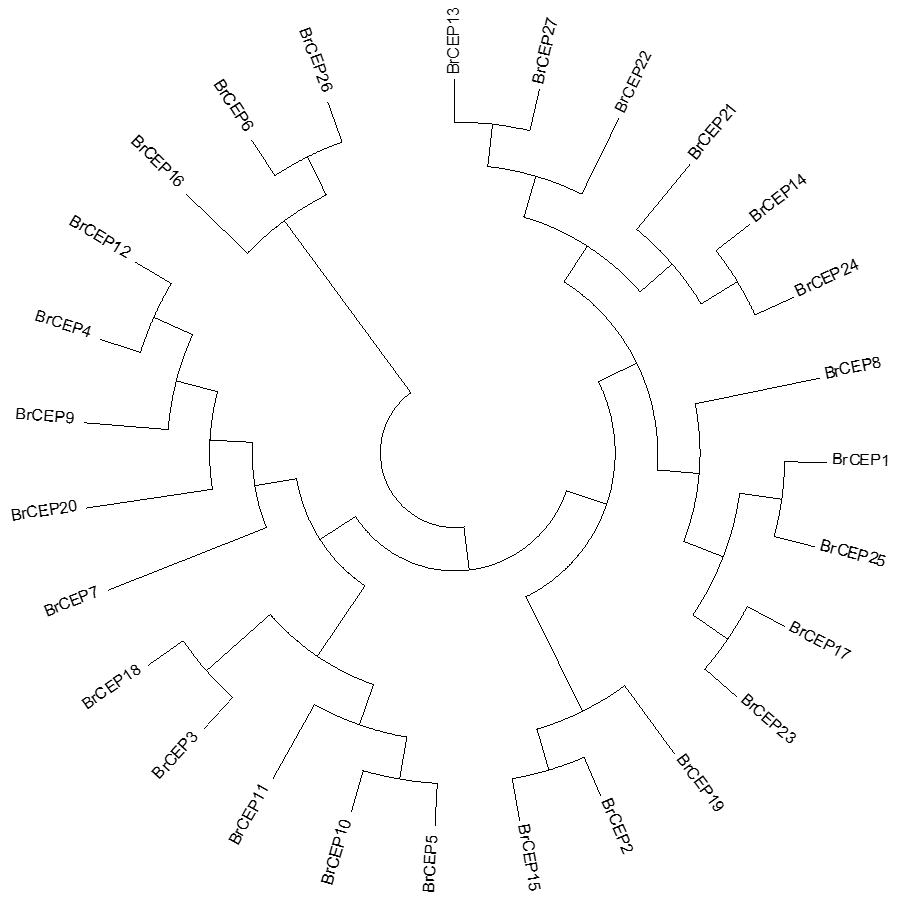
**

**Figure S2.** Phylogenetic tree of BrCEP proteins. The phylogenetic tree was build based on the full-length sequences of 27 BrCEP proteins with 1000 bootstrap replicates.


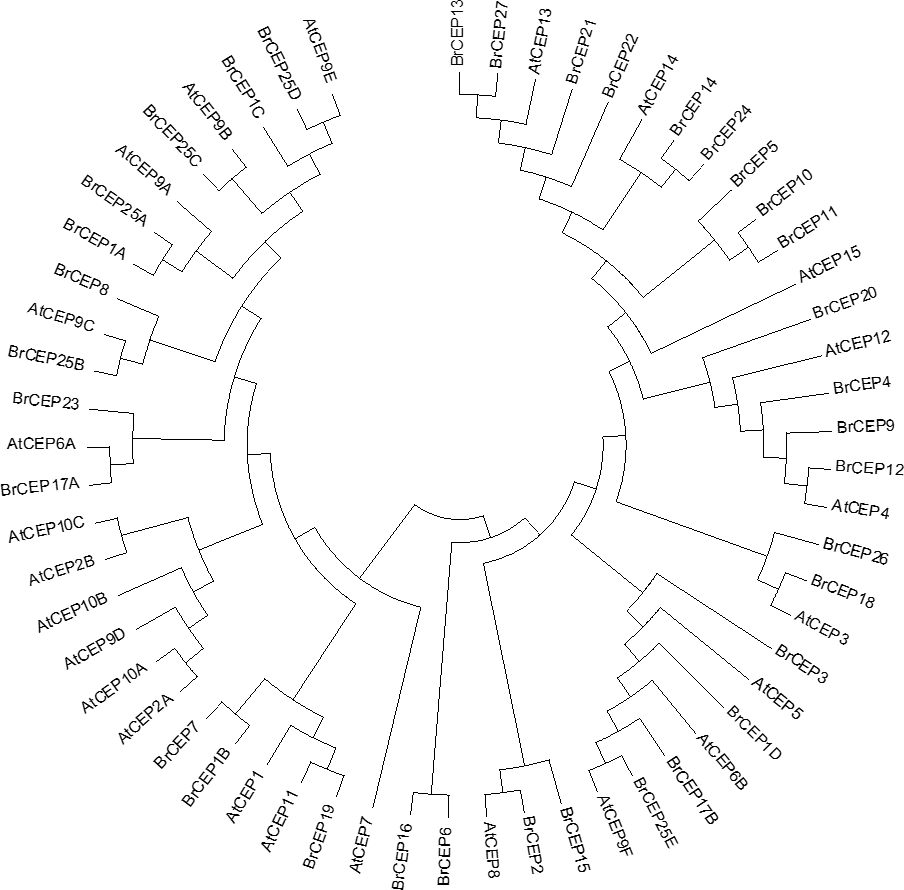


**Figure S3.** Phylogenetic tree of AtCEP and BrCEP proteins. The phylogenetic tree was build based on the CEP motifs of all BrCEP and AtCEP proteins with 1000 bootstrap replicates.

**
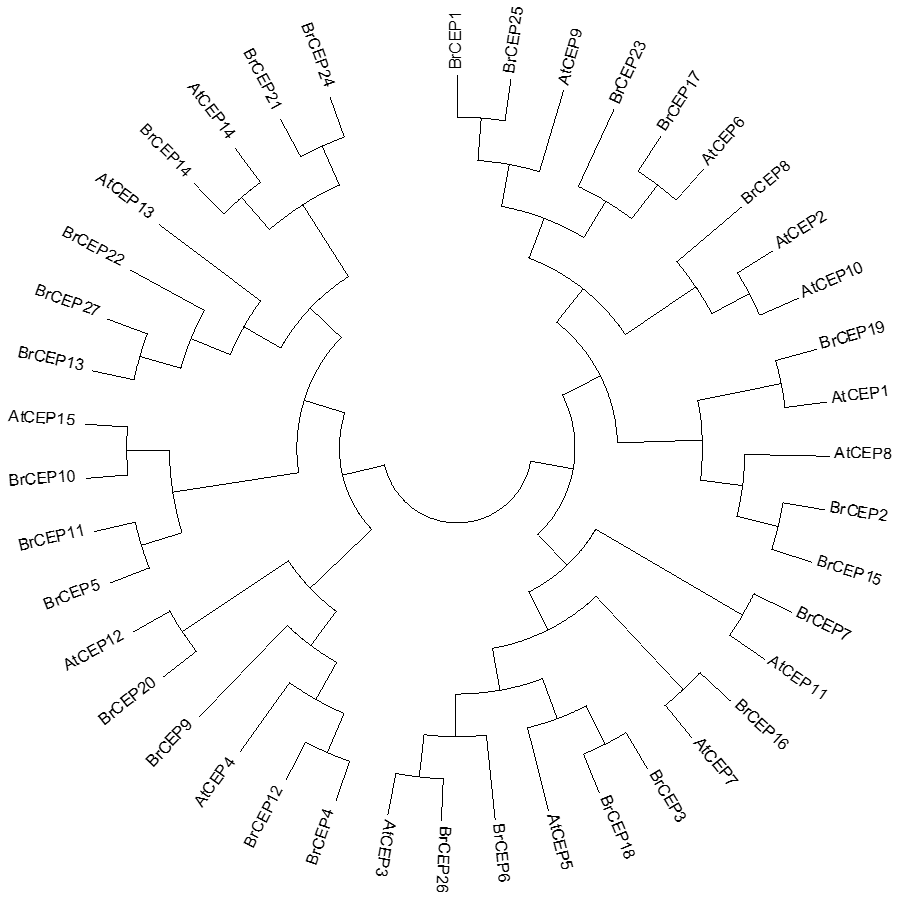
**

**Figure S4.** Phylogenetic tree of AtCEP and BrCEP proteins. The phylogenetic tree was build based on the full-length sequence of all BrCEP and AtCEP proteins with 1000 bootstrap replicates.

**
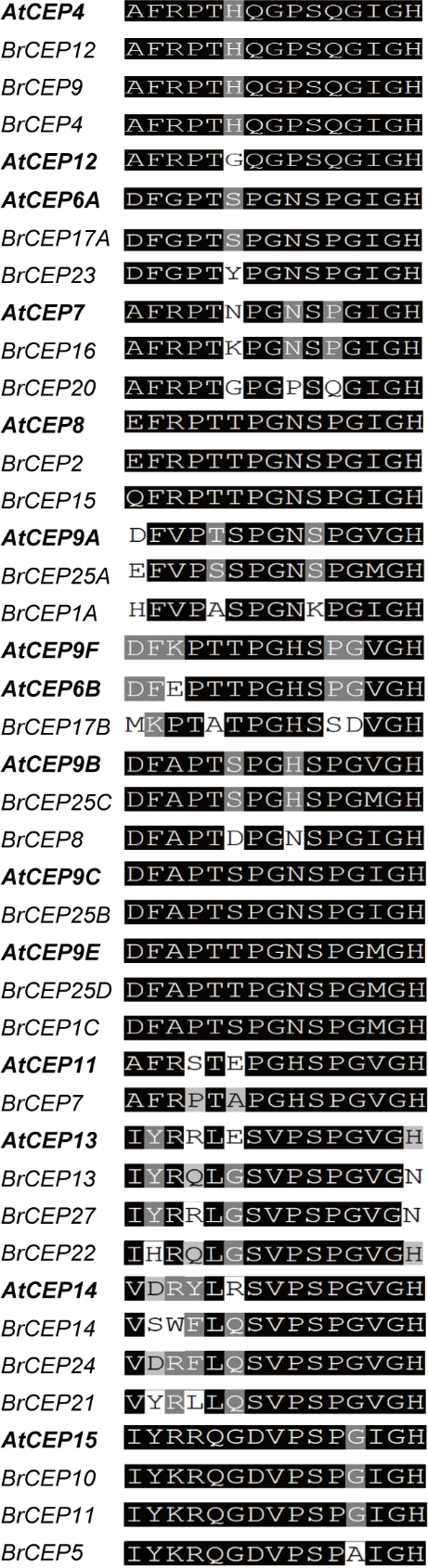
**

**Figure S5.** A list of the BrCEP and AtCEP proteins with a nearly identical CEP motifs.

**
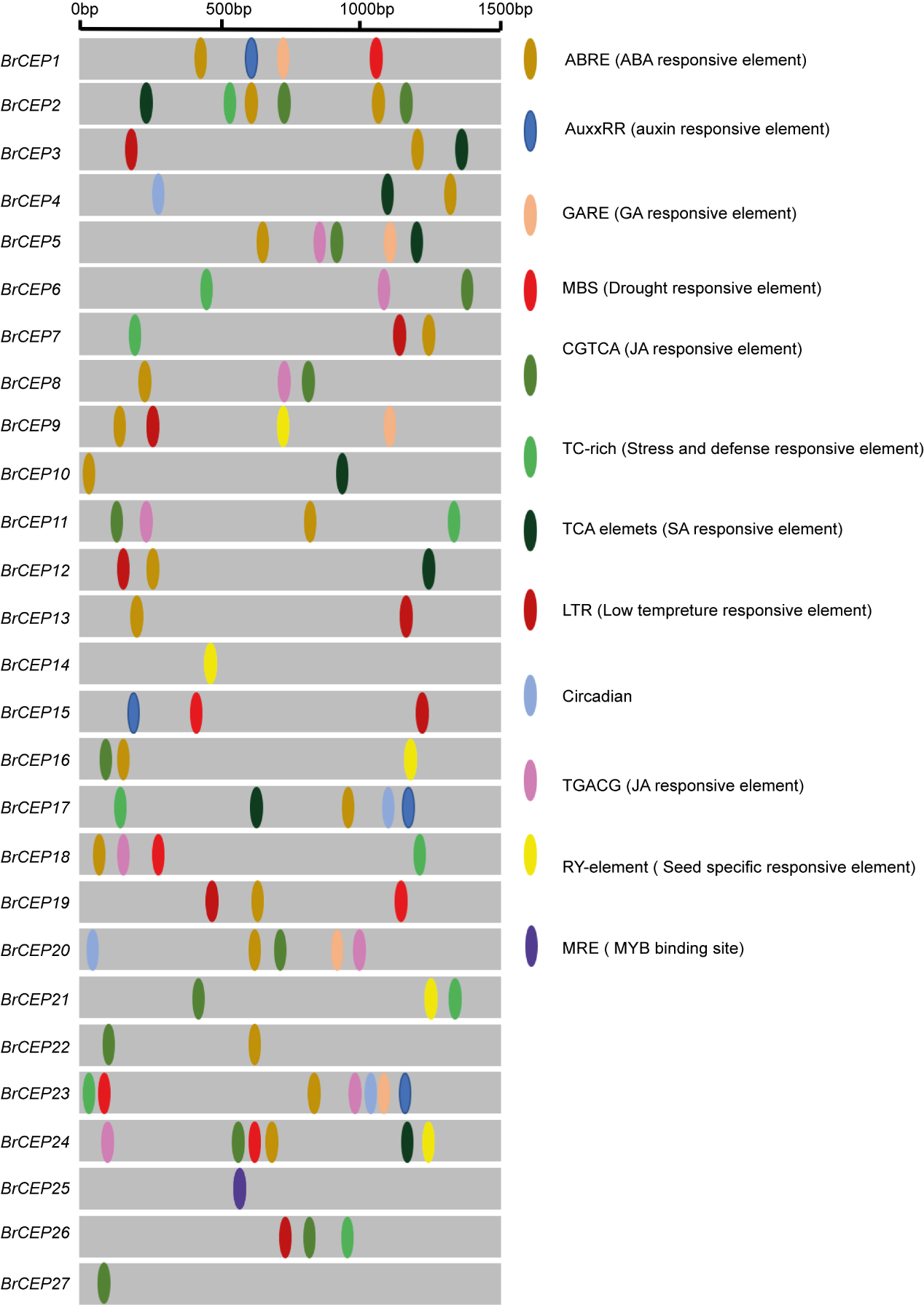
**

**Figure S6.** Prediction of the *cis*-acting regulatory elements in *BrCEP* gene promoters.


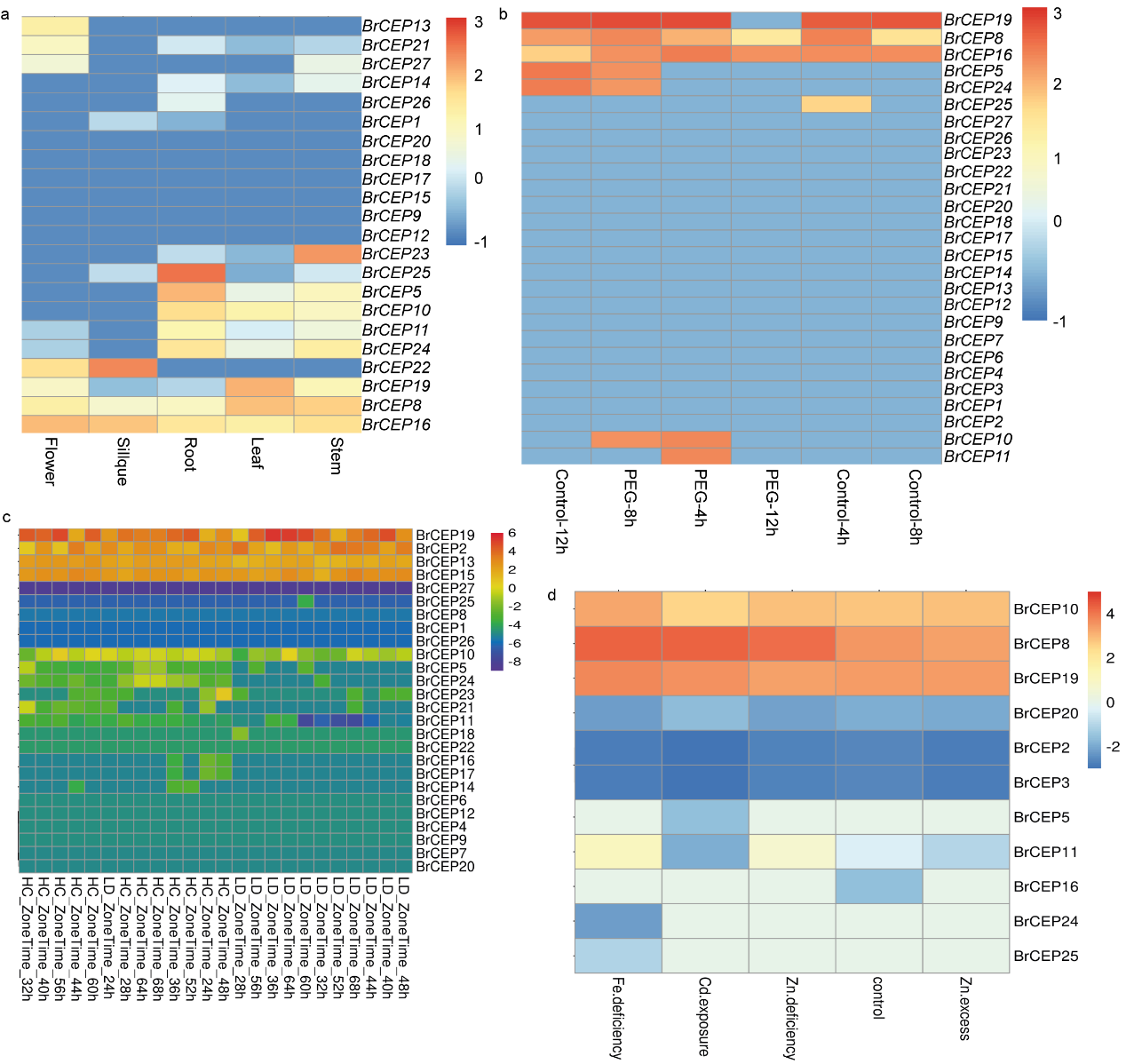


**Figure S7.** ***BrCEP*s respond to both developmental and environmental signals.** (a) Expression patterns of *BrCEP* genes in various tissues. The microarray data was download from GEO (GSE43245). (b) Expression patterns of *BrCEP* genes under drought stress. The microarray data was download from GEO (GSE73963). (c) *BrCEP* genes were induced by circadian rhythm. The microarray data was download from GEO (GSE123654). (d) *BrCEP* genes were induced by trace elements. The microarray data was download from GEO (GSE55264). Color scale represents log2 expression values.
